# Different paths, same destination, yet diverse effects: Antecedent configurations and performance implications of digital transformation in pharmaceutical manufacturing enterprises——Evidence from Chinese listed companies

**DOI:** 10.1371/journal.pone.0323130

**Published:** 2025-05-29

**Authors:** Bingxiao Shi, Chunlan Mou, Yiwen Zhang, Tiantian Cui

**Affiliations:** 1 School of Pharmacy, Shandong University of Traditional Chinese Medicine, Jinan, Shandong, China; 2 School of Health Management, Shandong University of Traditional Chinese Medicine, Jinan, Shandong, China; 3 Faculty of Economics and Management, Shandong Huayu University of Technology，Dezhou, Shandong, China; Indian Institute of Management Bodh Gaya, INDIA

## Abstract

The empowerment of the real economy through digital technology has emerged as a crucial force in driving enterprises toward high-quality development. Identifying effective strategies to accelerate the digital transformation of pharmaceutical manufacturing enterprises is essential for their upgrading and sustainable growth. This study adopts a configurational perspective, drawing on the Technology-Organization-Environment (TOE) theoretical framework. It integrates fuzzy-set qualitative comparative analysis (fsQCA) and propensity score matching (PSM) to explore the driving pathways of digital transformation in pharmaceutical manufacturing enterprises and further analyzes how distinct configurations influence firm performance. The research findings reveal three pathways to achieving a high level of digital transformation: “opportunity-perception”, “predatory-competition”, and “innovation-fueled”. However, there is significant variability in the impact of these three pathways on firm performance. These pathways collectively exemplify the concept of “different paths, same destination, yet diverse effects”. Sample enterprises exhibit inadequate investment in digital technology innovation; enterprise size, executives’ risk appetite and government support intensity emerge as significant facilitators of the digital transformation process within pharmaceutical manufacturing firms, while technological integration capabilities positively enhance the effectiveness of this transformation; An overreliance on the external environment will constrain the innovation autonomy of pharmaceutical manufacturing enterprises during digital transformation and hinder the scale effects of these enterprises. The conclusion offers pragmatic guidance for pharmaceutical manufacturing enterprises without digital technology innovation capabilities to select suitable digital transformation strategies to enhance enterprise performance.

## Introduction

In recent years, the emergence of digital technologies, including big data, artificial intelligence, and blockchain, has profoundly transformed the corporate development environment, leading to a relentless increase in data processing capabilities. This evolutionary shift highlights the ongoing transition of the human economy and society into a new era fundamentally rooted in the concept of “digitality”. The profound integration of digital technology with the actual economy is a crucial approach for enterprises to align with contemporary economic trends and bolster their competitiveness. Digital technology enables the pharmaceutical sector to optimize resource allocation, augment innovation capacity, enhance manufacturing and operational efficiency, and revolutionize therapeutic technologies to support personalized medicine [1,2]. However, in contrast to leading enterprises, the majority of pharmaceutical manufacturing enterprises typically encounter challenges such as inadequate capacity for new drug research and development, and the degree of informatization and automation in the production process remains at a stage characterized by isolated testing and localized implementation [3].

Scholars, both domestically and internationally, have conducted extensive explorations into the digital transformation of manufacturing enterprises and its associated outcomes. Existing research primarily explores general manufacturing enterprises across three dimensions: the external environment, internal resources and capabilities, and the characteristics and motivations of senior executives[4–7]. The digital transformation of pharmaceutical manufacturing enterprises leans more towards the innovation of production processes and business operations, and the digital transformation process itself is an innovative activity that combines complexity and dynamism [8]. To effectively analyze the multifaceted pathways of digital transformation in these enterprises and their corresponding outcomes, a comprehensive consideration of multiple factors is essential. Further, following the successful implementation of digital transformation, the ability to achieve the anticipated performance enhancement remains a primary concern for enterprises. Some experts have confirmed the beneficial impacts of digital transformation, including decreased manufacturing costs and enhanced technical innovation output [9]. Nevertheless, certain scholars contend that during digital transformation, enterprises encounter various challenges, including technological upgrades, organizational restructuring, and resource reallocation, with significant hidden costs that may result in a decline in short-term performance [10]. The digital transformation of pharmaceutical manufacturing encompasses the entire industrial chain, from research and development to production and clinical application. This process is highly complicated, and the efficacy of its transformation requires further verification.

Digital transformation involves the adoption and application of technology within manufacturing enterprises. The “Technology-Organization-Environment” (TOE) theoretical model serves as a comprehensive analytical framework based on technological application scenarios [11]. Therefore, grounded in the TOE theoretical model, this paper innovatively integrates Fuzzy-Set Qualitative Comparative Analysis (fsQCA) with Propensity Score Matching (PSM), utilizing samples from Chinese pharmaceutical manufacturing enterprises to refine the research into two progressively interrelated questions concerning transformation drivers and implementation effects. Firstly, the pathway question: Can various pathways, shaped by multiple influencing factors, all lead to successful digital transformation in pharmaceutical manufacturing enterprises? In other words, do “different paths” lead to “the same destination”? Secondly, the effectiveness question: Does digital transformation achieved through different pathways uniformly enhance firm performance? In essence, do “different paths” yield “the same outcomes”?

The marginal contributions of this paper are primarily evident in the following aspects: (1) It analyzes the intricate interplay among multiple factors influencing the digital transformation of pharmaceutical manufacturing enterprises and uncovers the diverse and multifaceted pathways that enable these enterprises to achieve high-level digital transformation. (2) This study assesses whether there are differences in performance enhancement across various driving pathways. It offers pragmatic guidance for pharmaceutical manufacturing enterprises without sufficient digital technology innovation capabilities to select an appropriate digital transformation strategy. (3) The integrated adoption of Fuzzy-Set Qualitative Comparative Analysis (fsQCA) and Propensity Score Matching (PSM) methodologies offers an exploratory approach that combines qualitative and quantitative methods, enhancing the depth and breadth of the research questions addressed, it is able to provide more comprehensive and scientifically sound recommendations and support for policy formulation and corporate management.

## Literature review and model construction

### Factors influencing digital transformation

The factors influencing the digital transformation of enterprises primarily involve organizational capabilities, top management, policy systems, industries, and regions. At the organizational level, dynamic capabilities facilitate the reintegration and reconfiguration of an enterprise’s innovation capabilities and resources, serving as an effective safeguard for enterprises to swiftly adapt to external environments and successfully implement transformation strategies [12]. Vial contends that organizational inertia can lead to path dependency, thereby hindering the digital transformation of enterprises [13]. Top management teams (TMTs) hold diverse perspectives on strategic planning and organizational structures [14]. During decision-making, the innovative service functions of TMTs significantly influence the execution of digital transformation initiatives within the organization [15]. Furthermore, leaders’ perceptual abilities and preferences for strategic goals also impact the digital transformation process [16]. Wu et al. argue that digital economy policies affect the technological innovation activities of microeconomic agents, with government fiscal expenditure on science and technology serving as a pivotal policy instrument that alleviates financial constraints and incentivizes enterprises’ digital transformation [17]. At the industry and regional levels, changes in market dynamics and competitive environments are primary drivers influencing enterprises’ propensity to adopt digital technologies. Advancements in industry technology and the evolution of consumer demands often motivate enterprises to harness digital technologies in pursuit of innovative and efficient solutions [18]. These multifaceted research findings provide theoretical support for the digital transformation of manufacturing enterprises. However, they lack specificity concerning pharmaceutical enterprises and seldom explore the synergistic effects of multiple factors driving the digital transformation of pharmaceutical manufacturing firms.

### Economic effects of digital transformation

The impact of digital transformation on enterprise performance can be observed in both positive and negative dimensions. From a proactive perspective, the introduction of digital technologies can expedite organizational innovation, resulting in tangible gains in innovative performance [19]. For instance, 3D printing technology has demonstrated transformative potential in the pharmaceutical sector through drug structure innovation, personalized medical solutions, production efficiency optimization, and therapeutic advancements. These technological breakthroughs fundamentally alter R&D paradigms and manufacturing processes, enabling precision medicine and cost-effective scalability [1].Guo et al. found that pharmaceutical manufacturing enterprises can leverage digital technologies to facilitate industrial structural upgrading. This advancement is specifically manifested in the digitalization of internal management systems, increased market expansion capabilities, improved productivity, and greater stability in quality control [2]. Conversely, from a challenge perspective, digital transformation represents a lengthy and dynamic process of technological innovation that is accompanied by hidden costs. Decision-making errors during this process can adversely affect an organization’s financial performance [20]. Additionally, the adoption of digital technologies may increase organizational management costs, leading to systemic imbalances that ultimately impede organizational performance [21]. Shackleton et al. argue that when digital technologies are incompatible with an organization’s core business operations, it can trigger conflicts between old and new resources, complicating operational processes [22]. Whether through case studies or empirical research, existing studies on the impact of digital transformation on performance have yet to reach a consensus.

The pharmaceutical industry, characterized by its stringent demands for specialization and safety, is further shaped in its digital transformation not only by technological advancements but also by various factors, including industry-specific characteristics, policy environments, and production processes. The international market share of China’s pharmaceutical industry is steadily rising, along with its global influence. In view of this, this study uses Chinese pharmaceutical manufacturing enterprises as a sample. Based on the three dimensions of “technology, organization, and environment,” It aims to identify the driving factors affecting digital transformation, explore the different transformation paths, and analyze the differentiated impacts of these paths on performance.

### Theoretical model construction

**Fig 1:**
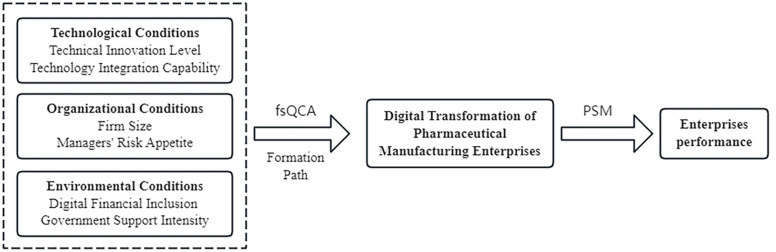
Framework of Theoretical Model.

This study explores how various factors interact and collectively drive this transformation across the three dimensions of technology, organization, and the digital environment. The theoretical model is illustrated in Fig 1.

Technological conditions encompass two sub-conditions: technological innovation capacity and technological integration capability. The level of technological innovation in enterprises is regarded as an agglomeration of capabilities forged through the implementation and support of technological innovation strategies, which involves an organization’s ability to perceive market needs, generate knowledge concepts, and bring them to commercialization [23]. The absence of essential technological innovation capabilities can have a detrimental impact on an organization’s digital transformation process. Furthermore, drawing upon the theory of technological innovation, technology integration can be conceptualized as the optimization and reconfiguration of technologies oriented towards manufacturing processes. Its essence lies in the innovation within the critical links of an enterprise’s value chain [24]. In the context of digital technologies facilitating high-quality industrial development, an enterprise’s core competitiveness increasingly depends on its ability to efficiently integrate resources [25]. In comparison to other industries, the implementation of technology integration in pharmaceutical manufacturing enterprises is particularly crucial, as it determines their ability to leverage efficient resource integration to economize on drug research and development costs as well as technology conversion costs.

Organizational conditions consist of two secondary factors: firm size and managers’ risk appetite. While technological advancements have the potential to “reinvent organizations,” they can also be influenced or even reshaped by countervailing organizational factors [26]. Research on the factors affecting the application of technological innovation typically emphasizes organizational and managerial levels, including the resources and capabilities of the organization and the structure of its executive team [27]. Among these factors, firm size and managers’ risk appetite are two of the most significant and quantifiable indicators. Drawing from the resource-based view, the adequacy of internal resources and the ability to transform heterogeneous resources are crucial for the effective implementation of digital transformation strategies [28]. According to Brickley et al., the level of risk appetite and support from corporate executives significantly influence the execution of major strategic initiatives and related projects. Managers with a higher level of risk appetite tend to increase capital expenditures and R&D investments, subsequently enhancing the efficiency of capital allocation and fostering a conducive environment for digital innovation activities [29].

Environmental conditions consist of two secondary factors: digital financial inclusion and government support intensity. The macro-environment can significantly affect the process of organizational transformation and upgrading. The notion of digital financial inclusion was initially introduced by the United Nations in 2005, and in 2016, the G20 established the G20 High-level Principles for Digital Financial Inclusion to advance global digital financial inclusion efforts. Digital financial inclusion refers to the application of emerging digital technologies to traditional financial activities, enhancing the optimal allocation of financial resources and improving the efficiency of financial service provision [30]. This inclusivity can effectively mitigate the property rights and scale biases present in traditional financial resource allocation, thereby increasing inclusivity and accessibility for disadvantaged financing groups, alleviating financing constraints, and promoting digital technological innovation activities [31]. The study conducted by Wu et al. Fiscal expenditure on science and technology possesses innovative targeting capabilities and demonstrates that fiscal science and technology expenditures can drive the digital transformation of enterprises by improving their financial health and enhancing their innovative capacity [17]. Pharmaceutical manufacturing enterprises that receive government subsidies and technological support can build a positive reputation, thereby attracting external investments. Additionally, such support allows these enterprises to diversify the risks associated with digital transformation and improve their innovation efficiency.

## Research methodology and data construction

### Research methodology

#### Qualitative comparative analysis (QCA).

This study employs fuzzy set qualitative comparative analysis (fsQCA) to investigate the various pathways influencing the digital transformation of pharmaceutical manufacturing enterprises, that is, do “different paths” lead to “the same destination”? By contrast with regression methods that isolate the effects of variables, this set-theoretic approach identifies causal configurations via Boolean minimization and incorporates counterfactual reasoning to clarify complex causal mechanisms [32]. QCA is categorized into three types based on data characteristics: crisp-sets QCA (csQCA), multi-value QCA (mvQCA), and fuzzy-sets QCA (fsQCA). In contrast to csQCA and mvQCA, which rely on binary or discrete categorical data, fsQCA permits continuous membership scores (ranging from 0 to 1) for both antecedent conditions and outcomes. effectively addressing the limitations of prior methods in capturing nuanced differences in condition intensities and outcome complexities.

Benefits of employing the fsQCA methodology. First, fsQCA could analyze the interdependence of antecedents and elucidate how various elements collaborate to facilitate digital transformation. Traditional regression-based methods frequently neglect nonlinear interactions and configuration effects. Second, fsQCA enables systematic cross-case comparisons across sample sizes to maintain a degree of external generalizability for the empirical results [33]. Third, fsQCA can analyze the condition configurations that result in the emergence and cessation of outcomes, offering pragmatic help for pharmaceutical businesses in selecting the suitable conversion pathway through exemplary case analysis.

#### Propensity Score Matching (PSM).

The Propensity Score Matching (PSM) approach is employed to investigate whether digital transformation, achieved through different paths, enhances the efficiency of enterprises, that is, whether “different paths” will produce “the same results”? PSM is a statistical technique commonly employed in observational studies within the field of economic management, the objective is to estimate the causal effect of a specific treatment while controlling for confounding variables [34]. In this study, PSM constructs a control group of pharmaceutical manufacturing enterprises with non-high-level digital transformation that closely resemble the main characteristics of the high-level transformation enterprises (treatment group). After matching, the control group serves as a ‘counterfactual’ for the treatment group, allowing for a comparison of the performance differences between the two groups following the high-level digital transformation. This methodology effectively addresses endogenous selection bias and unobserved heterogeneity while mitigating model specification errors and finite-sample biases inherent in conventional regression analyses.

#### Samples and data.

This research focuses on Chinese A-share listed pharmaceutical companies, utilizing data from 2017 to 2022. To ensure data quality, samples with ST, *ST status, abnormal financial data, or missing information were excluded, resulting in a final sample of 217 listed pharmaceutical companies. The data sources for this study include the China Stock Market & Accounting Research (CSMAR) Database, the WIND Database, the China National Research Data Service Platform (CNRDS), the China Science and Technology Statistical Yearbook, various regional statistical yearbooks, and the Digital Financial Inclusion Index published by the Institute of Digital Finance at Peking University.

#### Measurement of condition.

Digital transformation (DT): According to the “White Paper on China’s Digital Economy,” various industries in China intensified their exploration of novel organizational models integrating digital technologies in 2017. Consequently, the measurement interval for assessing the DT level of Chinese pharmaceutical manufacturing enterprises is defined as 2017–2021. As a critical corporate strategy, the characteristics of digital transformation are likely to be reflected in the comprehensive and directive annual reports of enterprises, where the terminology used serves as a mirror, reflecting both the strategic orientation of the enterprise and its aspirations for the future [7,35]. Therefore, extracting the frequency of words related to “digital transformation” from the annual reports of pharmaceutical-listed companies to delineate their degree of digital transformation represents a scientific and feasible approach. This study employs textual analysis to quantify the degree of DT within the pharmaceutical manufacturing sector. Given the right-skewed distribution of the data, a natural logarithm transformation is applied after adding 1 to ensure statistical appropriateness.

Technical Innovation Level (RD): Sales revenue serves as a tangible indicator of the economic value generated by research and development (R&D) investments. This study measures the level of technological innovation by adopting the ratio of R&D investment to sales revenue for sample firms in 2020 [36].

Technology Integration Capability (RDP): Research personnel endowed with heterogeneous characteristics represent the most creative form of human capital. The distinctive innovative behaviors and performance exhibited by heterogeneous R&D personnel constitute a crucial driving force for enterprises to effectively integrate and utilize both novel and existing technologies. Consequently, this paper uses the proportion of internal R&D personnel within pharmaceutical manufacturing enterprises in 2020 as a proxy variable for technology integration capability [37].

Firm Size (SIZE): Firm size is measured by the natural logarithm of the total assets of pharmaceutical manufacturing enterprises at the end of 2020 [38].

Managers’ Risk Preference (MRP): Risk preference encapsulates the attitude of decision-makers toward uncertain risks during strategic decision-making, which further externalizes investment decisions. This paper adopts the ratio of risky assets to total assets in 2020 as the measurement for risk preference [39].

Digital Financial Inclusion (DFI): The DFI is sourced from the “Peking University Digital Financial Inclusion Index,” published by the Institute of Digital Finance, Peking University, in 2020. The index encompasses comprehensive data from 31 provinces, 337 prefecture-level cities, and their respective county-level regions across China.

Government Support Intensity (GST): Given the limitations of the absolute indicators of government financial expenditure on science and technology, a relative indicator of local government financial expenditure on science and technology is adopted for measurement. The intensity of government support is represented by the ratio of local fiscal expenditure on science and technology to local general public budget revenue in 2020 [17].

The conditional measurements are shown in Table 1.

**Table 1:** Measurement of condition.

	Condition Variable	Variable Measurement
**Outcome Variable**	DT	Employs textual analysis
**Technological Conditions**	RD	R&D investment/sales revenue
	RDP	Proportion of internal R&D personnel within enterprises
**Organizational Conditions**	SIZE	LN (Total assets at year-end)
	MRP	Risky assets/total assets
**Environmental Conditions**	DFI	Peking University Digital Financial Inclusion Index
	GST	Intensity of fiscal expenditure on science and technology

## Data analysis and empirical results

### Variable calibration

The process of assigning cases to set affiliation scores is called calibration [40]. In this paper, a direct calibration method is employed to transform raw data into fuzzy set affiliation scores ranging from 0 to 1 [41]. The 0.95, 0.5, and 0.05 quantile values of the outcome and condition variables serve as qualitative anchors for full affiliation, crossover points, and full non-affiliation, respectively [42,43]. Furthermore, in order to avoid the issue of unknown grouping attribution associated with a pooled affiliation score of 0.50 for the case samples, a constant of 0.001 is added to the 0.5 affiliation. The calibration information for each antecedent condition and outcome is presented in Table 2.

**Table 2:** Variable calibration.

	Conditional Variable	Full Affiliation	Crossover	Incomplete Affiliation
**Outcome Variable**	DT	5.220	3.989	2.532
**Technological Conditions**	RD	25.062	5.610	1.748
	RDP	33.392	15.060	4.088
**Organizational Conditions**	SIZE	23.863	22.058	20.609
	MRP	0.313	0.046	0.001
**Environmental Conditions**	DFI	5.771	5.681	5.505
	GST	0.130	0.058	0.016

### Univariate analysis of necessity

The necessity of individual conditions was examined using the fsQCA method, and the results, as presented in Table 3, indicate that all necessity scores are below 0.9. This finding suggests that there are no necessary conditions for achieving either high or non-high levels of digital transformation in the pharmaceutical manufacturing industry.

**Table 3:** Necessity analysis.

Conditional Variable	High Level of Digital Transformation		Non-High Level of Digital Transformation	
	Consistency	Coverage	Consistency	Coverage
**RD**	0.546	0.614	0.640	0.719
**~RD**	0.751	0.677	0.657	0.591
**RDP**	0.580	0.607	0.675	0.705
**~RDP**	0.718	0.689	0.624	0.597
**SIZE**	0.708	0.738	0.550	0.572
**~SIZE**	0.590	0.568	0.748	0.718
**MRP**	0.558	0.633	0.581	0.658
**~MRP**	0.699	0.625	0.676	0.604
**DFI**	0.636	0.586	0.714	0.657
**~DFI**	0.628	0.688	0.550	0.601
**GST**	0.616	0.660	0.622	0.665
**~GST**	0.687	0.646	0.682	0.639

Note: “~” means “not”.

### Adequacy analysis of conditional configuration

The sufficiency of configurations is analyzed using a truth table algorithm to determine the relationship between relevant configurations and outcomes. A configuration is considered sufficient for an outcome when the consistency threshold is at least 0.75 [44]. Based on the consistency score gaps presented in the truth table and the sample size, the consistency threshold is set at 0.8, while the frequency threshold is established at 1.

Table 4 presents the configurations of conditions that enable high-level digital transformation in pharmaceutical manufacturing enterprises. On an aggregate level, the overall consistency of these five configurations is approximately 0.86, surpassing the generally accepted threshold of 0.80, while the overall solution coverage is around 0.53. All five configurations serve as sufficient conditions for achieving high-level digital transformation within pharmaceutical manufacturing enterprises. Upon further inductive analysis, it is noted that Configuration 1a resembles Configuration 1b, and Configuration 2a mirrors Configuration 2b. Based on these findings, the following three pathways to high-level digital transformation are summarized:

**Table 4:** Configuration for achieving a high level of digital transformation.

Conditional Variable	Opportunity-Perception Type		Predatory-Competition Type		Innovation-Fueled Type
	1a	1b	2a	2b	3a
**RD**		⨂	⨂		⨂
**RDP**			⨂	⨂	●
**SIZE**	●	●	●	●	●
**MRP**	●	●		⨂	●
**DFI**	⨂	⨂		●	
**GST**	⨂		●	●	⨂
**Consistency**	0.9118	0.9144	0.8745	0.8871	0.8873
**Original Coverage**	0.3018	0.2961	0.3895	0.3260	0.2479
**Unique Coverage**	0.0322	0.0047	0.0364	0.0145	0.0087
**Consistency of Solution**	0.8625				
**Coverage of Solution**	0.5332				

Note: ● and ● indicate that core conditions are present, and core conditions are missing; ⨂ and ⨂ indicate that marginal conditions are present and marginal conditions are missing, respectively.

(1) Path 1 (SIZE* MRP) comprises enterprises with large-scale operations and executives who exhibit a high level of risk appetite, although these firms are characterized by relatively low levels of digital financial inclusion and government support. When faced with a limited external supply of resources, large pharmaceutical manufacturing enterprises leverage their substantial resource base to support their digital transformation initiatives. The high-risk appetite among executives enables them to swiftly capture market dynamics and formulate responsive strategies, thereby reinforcing the strategic impetus for digital transformation. Consequently, this path is designated as the “opportunity-perception” type. Taking Yunnan Baiyao corporation as an example under this path, this enterprise has consistently advanced digital and intelligence-driven pharmaceutical innovations, fully integrating digital transformation into its corporate culture, systems, production framework, and organizational structure. By enhancing business management intelligence through digital means, Yunnan Baiyao has been recognized as a benchmark enterprise in the biomedical industry for its digital transformation efforts in China.
(2) Path 2 (SIZE*GST*DFI) represents large-scale pharmaceutical manufacturing enterprises that leverage government support and digital financial inclusion to achieve digital transformation. This model illustrates how enterprises utilize external resources within a favorable innovation environment to pursue transformation. By capitalizing on their advantageous status, large-scale pharmaceutical companies secure greater government innovation subsidies and digital financial inclusion support, thereby alleviating financing constraints and facilitating the effective implementation of digital transformation activities. Consequently, this pathway is designated as the “Predatory-Competition” type. For instance, the well-established Jiuzhitang Corporation exemplifies this path. With the support of government departments, the company developed the “Intelligent Factory Integrated Application New Model for Traditional Chinese Medicine Solid Dosage Forms” in 2020, implementing comprehensive intelligent control over the production process for traditional Chinese medicine solid dosage forms. Furthermore, Jiuzhitang has actively expanded its new “Internet + Traditional Chinese Medicine Medical Treatment” online consultation model, emerging as a notable example of digital technology innovation in the pharmaceutical manufacturing industry.
(3) Path 3 (SIZE*MRP*RDP) represents a configuration where large-scale enterprises and executives play central roles characterized by a high level of risk appetite, while technological integration capabilities serve as supplementary and complementary conditions. This path indicates that when enterprises are strongly motivated to implement digital transformation strategies and possess a solid resource base, emphasizing the enhancement of digital technology integration capabilities can help unleash production efficiency and accelerate the digital transformation process. Therefore, this pathway is designated as the “Innovation-Fueled” type. For instance, Huaxi Biotech Corporation exemplifies this approach. As a platform enterprise spanning the entire biotechnology industry chain, Huaxi has collaborated with the UDS process industry team to explore and develop a pathway toward digital transformation. By implementing FineBI and establishing a data mid-platform operation center, Huaxi has created a “Bio-Smart Factory” that integrates informatization, intellectualization, and datafication.

### Robustness test

To conduct robustness checks, adjustments were made to the consistency threshold and case frequency. Following the approach of Ordanini et al., the consistency threshold was increased from 0.80 to 0.85 [45], and the results are presented in Table 5. Additionally, the case frequency was raised from 1 to 2, yielding three configurations that are subsets of the existing configurations. A comprehensive comparison revealed that the adjusted configurations exhibited generally consistent results, indicating the robustness of the research findings.

**Table 5:** Robustness test results.

conditional variable	opportunity-perception type		predatory-competition type		innovation-fueled type
	1a	1b	2a	2b	3a
**RD**		⨂	⨂		⨂
**RDP**			⨂	⨂	●
**SIZE**	●	●	●	●	●
**MRP**	●	●		⨂	●
**DFI**	⨂	⨂		●	
**GST**	⨂		●	●	⨂
**Consistency**	0.9118	0.9144	0.8746	0.8871	0.8873
**Original Coverage**	0.3018	0.2961	0.3900	0.3260	0.2480
**Unique Coverage**	0.0322	0.0047	0.0364	0.0145	0.0087
**Consistency Of Solution**	0.8626				
**Coverage Of Solution**	0.5332				

Note: ● and ● indicate that core conditions are present, and core conditions are missing; ⨂ and ⨂ indicate that marginal conditions are present and marginal conditions are missing, respectively

## Impact of different configurations on the performance of pharmaceutical manufacturing enterprises

To explore the differentiated impacts of various digital transformation pathways on corporate performance, this study employs the Propensity Score Matching (PSM) method to investigate further whether “different paths” lead to “similar effects.” This approach aims to elucidate the complex relationship between the diverse digital transformation strategies adopted by pharmaceutical manufacturing enterprises and their corporate performance.

### Calculating the propensity score for High-Level digital transformation

Firstly, the propensity scores for Chinese pharmaceutical manufacturing enterprises to implement various types of high-level digital transformation are estimated. A Logit regression model is utilized for prediction, with the implementation of opportunity-perception transformation, predatory-competition transformation, and innovation-fueled transformation serving as the dependent variables. Drawing on relevant research [9,46], explanatory variables are selected from several dimensions, including firm characteristics, financial risks, and governance capabilities. Specifically, the following variables are included: (1) Enterprise age (Age): The natural logarithm of the number of years since the establishment of the pharmaceutical listed company up to the observation period. (2) Enterprise size (Size): The natural logarithm of the total assets of the enterprise. (3) Leverage Ratio (Lev): The ratio of total liabilities to total assets of the enterprise. (4) Liquidity Ratio (Liq): The ratio of total current assets to total assets of the enterprise. (5) Asset Turnover (ATO): The ratio of operating income to the ending balance of total assets of the enterprise. (6) Shareholding Concentration (First): The percentage of shares held by major shareholders. (7) Board Size (Board): The natural logarithm of the number of directors on the board of the enterprise. The results of the Logit regression are presented in Table 6.

**Table 6:** Logit model regression results for propensity score.

	Opportunity-Perception Type	Predatory-Competition Type	Innovation-Fueled Type
**Age**	1.735 (1.42)	3.556^**^ (2.54)	1.478 (0.62)
**Size**	0.920^***^ (3.31)	0.929^***^ (3.37)	0.116 (0.20)
**Lev**	-0.030 (-1.63)	-0.021 (-1.20)	-0.017 (-0.36)
**Liq**	0.893 (0.53)	-1.643 (-0.92)	1.930 (0.49)
**ATO**	-0.618 (-0.45)	3.177^**^ (2.31)	-1.535 (-0.45)
**First**	0.005 (0.29)	-0.011 (0.60)	-0.003 (-0.07)
**Board**	-0.663 (-0.49)	1.121 (0.78)	7.097^*^ (1.93)
**Constant** **(math)**	-26.387^***^ (-4.31)	-37.108^***^ (-5.25)	-26.617^*^ (-1.78)
**Pseudo-R2**	0.1483	0.2525	0.1505
**N**	217	217	217

Note:

*,

**,

***indicate statistical significance at the 10 percent, 5 percent, and 1 percent levels, respectively.

### Sample matching effect

**Fig 2:**
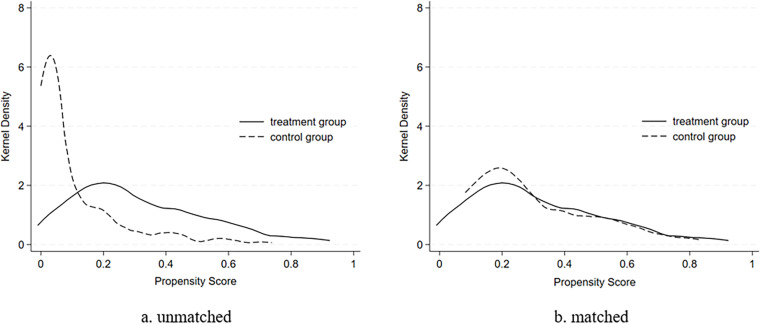
The Kernel Density Estimation Plots of The Treatment and Control Groups.

Sample matching was performed using the propensity scores of firms that achieved high-level digital transformation through different pathways. Using the predatory-competition type as an example, the nearest neighbor matching method (at a 1:2 ratio) was applied. Table 7 presents the balance test of matching variables before and after matching. Fig 2 displays the kernel density estimation plots of the treatment and control groups before and after matching. Post-matching, the bias of all control variables was significantly reduced, and the differences between the treatment and control groups became statistically insignificant (P > 0.476). The LR-Chi2 value of the matching model decreased from 45.21 to 0.82, while the corresponding p-value increased from 0.000 to 0.997, indicating a more favorable match.

**Table 7:** PSM equilibrium tests for predatory competition models.

Variable Matching	Treatment Group	Control Group	Bias	|Bias|	t	p>|t|	V(T)/V(C)
**Age(U)**	3.2457	3.0855	83.2		3.46	0.001	0.24*
**Age(M)**	3.2457	3.2209	12.9	84.5	0.72	0.476	0.71
**Size(U)**	23.115	22.074	117.5		5.76	0.000	0.89
**Size(M)**	23.115	23.074	4.7	96.0	0.19	0.853	1.07
**Lev(U)**	31.735	28.890	19.9		0.97	0.335	0.84
**Lev(M)**	31.735	31.364	2.6	87.0	0.10	0.922	0.82
**Lip(U)**	0.5561	0.5362	12.9		0.62	0.539	0.74
**Lip(M)**	0.5561	0.5574	-0.8	93.6	-0.03	0.976	0.64
**ATO(U)**	0.5977	0.4779	63.4		3.11	0.002	0.89
**ATO(M)**	0.5977	0.6254	-14.6	76.9	-0.55	0.583	0.87
**First(U)**	34.014	30.316	30.3		1.51	0.134	0.95
**First(M)**	34.014	33.96	0.4	98.6	0.02	0.987	0.86
**Board(U)**	2.1931	2.0939	61.3		2.73	0.007	0.46*
**Board(M)**	2.1931	2.2012	-5.0	91.8	-0.16	0.857	0.28*

Note: U(unmatched), M(matched)

### Analysis of the impact of different types of High-Level digital transformation on business performance

Considering the inherent time lag in the effects of digital transformation on firm performance, the Return on Assets (ROA) at time t + 1 is selected as the metric for evaluating corporate performance. To estimate the Average Treatment Effect on the Treated (ATT), the nearest neighbor matching approach with a 1:2 ratio is employed. Robustness checks are conducted using both radius matching with a caliper of R = 0.02 and kernel matching methods. The results of this analysis are presented in Table 8.

**Table 8:** Analysis of the impact of different models of high-level digital transformation on the subsequent performance of pharmaceutical manufacturing enterprises (PSM).

Matching method	opportunity-perception type		predatory-competition type		innovation-fueled type	
	ATT	T	ATT	T	ATT	T
**Unmatched**	0.0320	1.60	-0.0197	-1.06	0.0537	1.15
**Close neighbor** **matching**	0.0194	1.13	-0.0373	-1.97^**^	0.1084	1.97^**^
**Radius** **matching**	0.0242	1.40	-0.0527	-2.92^***^	0.0808	1.67^*^
**Kernel** **matching**	0.0212	1.25	-0.0344	-2.05^**^	0.0834	1.73^*^

Note:

*,

**,

***indicate statistical significance at the 10 percent, 5 percent, and 1 percent levels, respectively.

Following the nearest neighbor matching process, pharmaceutical manufacturing enterprises that embraced the opportunity-perception pathway exhibited high-level subsequent performance following the implementation of advanced digital transformation relative to the control group; nonetheless, the results lacked statistical significance (ATT = 0.0194, p > 0.10). The scenario illustrates that while senior managers have captured market dynamics in a timely manner and used their deep resources to achieve digital transformation, numerous issues persist. First, management perceives digital transformation merely as a technical tool and emphasizes short-term outcomes, so that it is difficult to reap the long-term benefits of digital transformation for the enterprise. Secondly, during the transition process, enterprises encounter a deficiency of digital talent who possess both technological proficiency and business acumen, together with digital capabilities and inventive thinking. As a result, enterprises fall into the dilemma of blind digital deployment in an isolated way, which makes it difficult for enterprises to obtain the corresponding value return from digital investment. Furthermore, the organization faces problems like an antiquated system, a conventional management structure, and convoluted processes, which hinder the integration of digital technologies with current resources, resulting in minimal enhancement of enterprise performance.

Under the predatory-competition pathway, the subsequent performance of pharmaceutical manufacturing enterprises implementing high-level digital transformation is significantly lower than that of the control group (ATT = -0.0373, p < 0.10). This path cannot substantially enhance performance. In the domain of enterprise digital transformation research, a nonlinear “inverted U-shaped” correlation exists between digital transformation and enterprise performance, indicating that both excessive and insufficient digital transformation might adversely affect performance enhancement [47]. Under this digital transformation path, pharmaceutical manufacturing enterprises encounter numerous challenges along this digital transformation trajectory. Initially, there exists an imbalance in resource distribution. Despite advancements in digital financial inclusion and governmental investment in technology enhancing access to financing, the inherent lack of strategic flexibility within enterprises has resulted in a significant imbalance in resource allocation, ultimately undermining the potential efficacy of digital technology. Secondly, there is a phenomenon of blind investment. Some enterprises lack clear digital transformation plans and goals, and it is difficult to accurately evaluate and optimize the implementation of digital transformation. In addition, without fully combining the actual needs of the business, enterprises are driven by the external environment to introduce a large number of new technologies and funds to carry out digital transformation, which is easy to cause problems such as incompatible business systems. Thirdly, inadequate investment in technical innovation. The deficiency in digital technology innovation and integration capabilities may obstruct an effective amalgamation of digital technology with production and operations, hence impeding enhancements in corporate performance.

In contrast, pharmaceutical manufacturing enterprises that adopt the innovation-fueled pathway demonstrate significantly higher subsequent performance after implementing high-level digital transformation compared to those in the control group (ATT = 0.1084, p < 0.10). In contrast to the opportunity-sensing path, digital integration capacity is significant and positively influences performance. At first, in terms of resource allocation optimization, digital technologists use their expertise to introduce advanced and efficient new-generation digital technologies, improve existing products and services, expedite innovative research and development, reduce drug development timelines, and augment production capacity. Simultaneously, digital technologists facilitate the adoption of innovative services or business models, enhance management efficiency, minimize redundant expenses, and augment market share and performance metrics. Moreover, digital technicians apply digital platforms to streamline information exchange and activity coordination within organizations, augment organizational agility, and promote employee motivation and productivity. Furthermore, digital technicians optimize business processes and improve production by utilizing digital technology, hence positively influencing enterprise performance.

It can be seen that pharmaceutical manufacturing enterprises that achieve digital transformation through an innovation-driven path can significantly improve corporate performance. Huaxi Biotechnology Company exemplifies successful outcomes in digital transformation. In the production and manufacturing sector, adhering to Industry 4.0 standards to establish intelligent “black light factories” achieves the whole process of production information, intelligence, and unmanned operations, effectively saving costs and improving the production accuracy and yield. In technological research and development, AI is utilized in synthetic biology and integrated with high-throughput screening technologies to enhance project development efficiency and product quality. In terms of digital technical talents, Huaxi Biological’s effective management and innovation of the whole industrial chain have improved the company’s overall operational efficiency and management level and won the “China Talent Management Benchmarking Enterprise Award.” Specifically, Huaxi Biology collaborates with academic institutions to produce a cadre of professionals equipped with digital competencies and innovative mindsets, thereby ensuring a talent foundation for the digital advancement of firms. At the same time, the talent training system is continuously enhanced to assist employees in advancing their professional skills and business acumen in the digital domain, thereby addressing the requirements of organizations’ digital transformation. Furthermore, enhance and refine human resource management, implement sub-sequence management, and develop a three-channel career model comprising M (management position), P (expert position), and T (technical position) to offer a varied and multi-faceted career development pathway for digital talents across diverse levels and professions, thereby invigorating the digital transformation of enterprises. Consequently, pharmaceutical manufacturing enterprises should improve the ability of technology integration, increase the introduction of digital technology and digital talent investment, and bolster market competitiveness and operational efficiency.

### Conclusion and enlightenment

This paper, grounded in the Technology-Organization-Environment (TOE) theoretical framework, employs the fuzzy-set Qualitative Comparative Analysis (fsQCA) methodology to explore the configurational effects of technological, organizational, and environmental factors on the digital transformation of Chinese pharmaceutical manufacturing enterprises. Additionally, the Propensity Score Matching (PSM) method is utilized to examine the differential impacts of digital transformation, driven by distinct models, on firm performance. The key findings are as follows: (1) The validation outcomes indicate that the digital transformation of these enterprises is not solely facilitated by a singular condition; rather, it requires a concerted effort involving multiple factors, including technological innovations, organizational structural adjustments, and support from the external digital environment, to achieve the anticipated outcomes. (2) The achievement of high-level digital transformation in pharmaceutical manufacturing enterprises can be categorized into four equivalent configurations, which can be consolidated into three distinct pathways: opportunity-perception, predatory-competition, and innovation-fueled. The impact of the three pathways on the performance of pharmaceutical enterprises is different, illustrating “diverse paths leading to similar goals, yet with varying effects.” (3) Enterprise size, executives’ risk appetite, and government support intensity are the primary determinants for pharmaceutical manufacturing enterprises to achieve high-level digital transformation. Neglecting technological integration capabilities and excessively depending on the external environment may hinder firms’ independent innovation capacity, which is not conducive to exerting the scale effect of enterprises and thus affecting the improvement of performance.

Based on the above research, we provide the following implications.

Initially, pay attention to the integrity and system of the digital transformation of pharmaceutical manufacturing enterprises. Pharmaceutical manufacturing enterprises with insufficient digital technology innovation started late in digital transformation, mostly staying at the sales and management level, and failed to fully expand to the production level. Consequently, enterprises should design and construct a set of technology-enabling systems that integrate with the current drug production, organizational structure, and management process efficiently, so as to realize the update and iteration of the whole chain of pharmaceutical manufacturing enterprises. Furthermore, enterprise managers should start from a holistic perspective, dynamically monitor and optimize and adjust the process of digital transformation in real time, and avoid potential risks in production, finance, and management. Secondly, reshape the strategic system of enterprises and seize the development opportunities of the digital economy. The decisions of pharmaceutical manufacturing enterprises are typically influenced by external factors, including the market and government regulations. Despite the global promotion and implementation of digital financial inclusion, disparities exist in its execution among countries. China has attained a preeminent position in this domain globally. Consequently, leveraging China’s experience, nations should prioritize the facilitative and promotional function of digital inclusive finance in the application of digital technology and financing for pharmaceutical manufacturing enterprises while expediting the development of financing infrastructure and the establishment of laws and regulations governing digital inclusive finance. Simultaneously, governments must provide macroeconomic direction and financial assistance to foster a conducive digital transformation environment for pharmaceutical manufacturing firms lacking digital technology innovation capabilities. For enterprises, they should fully leverage external support, increase organizational innovation potential, and enhance source innovation and achievement transformation. Managers must focus on fostering digital thinking, augmenting investments and support for digital transformation, and enhancing their strategic decision-making capabilities in this domain.

Third, enhance the degree of digital technology integration and foster autonomous innovation capabilities. Pharmaceutical manufacturing enterprises lacking in digital technology innovation should closely monitor governmental guidance and industry trends, enhance investment in technological innovation, and proactively recruit digital talent. During the digital transformation of pharmaceutical manufacturing enterprises, digital technicians must offer robust technical support and strategic guidance to facilitate efficient research and development and bolster the core competitiveness of these firms. Simultaneously, the introduction of high-end medical equipment strengthens the integration of intelligent systems in each link, establishing a comprehensive intelligent and automated control system throughout the production process. In operational management, enterprises should establish a data platform to systematically integrate the operational management information system with drug research and development, production, sales, and user service, and deeply optimize the intelligent decision-making processes in enterprise production and operational management. Marketing initiatives should focus on developing integrated intelligent marketing channels and communication platforms to address the trend of varied medicine purchasing avenues and the individualized requirements of patients.

## Supporting information

S1 Data (XLSX)
